# Structural Response Prediction of Thin-Walled Additively Manufactured Parts Considering Orthotropy, Thickness Dependency and Scatter

**DOI:** 10.3390/ma14092463

**Published:** 2021-05-10

**Authors:** Sigfrid-Laurin Sindinger, David Marschall, Christoph Kralovec, Martin Schagerl

**Affiliations:** 1 Institute of Structural Lightweight Design, Johannes Kepler University Linz, 4040 Linz, Austria; christoph.kralovec@jku.at (C.K.); martin.schagerl@jku.at (M.S.); 2 Christian Doppler Laboratory for Structural Strength Control of Lightweight Constructions, 4040 Linz, Austria; 3 KTM E-TECHNOLOGIES GmbH, 5081 Anif, Austria; David.marschall@ktm.com

**Keywords:** additive manufacturing, carbon-fiber-reinforced polymers, thin-walled structures, orthotropy, thickness dependency, experimental testing, material modeling, property mapping, structural response prediction, stochastic finite element analysis

## Abstract

Besides the design freedom offered by additive manufacturing, another asset lies within its potential to accelerate product development processes by rapid fabrication of functional prototypes. The premise to fully exploit this benefit for lightweight design is the accurate structural response prediction prior to part production. However, the peculiar material behavior, characterized by anisotropy, thickness dependency and scatter, still constitutes a major challenge. Hence, a modeling approach for finite element analysis that accounts for this inhomogeneous behavior is developed by example of laser-sintered short-fiber-reinforced polyamide 12. Orthotropic and thickness-dependent Young’s moduli and Poisson’s ratios were determined via quasi-static tensile tests. Thereof, material models were generated and implemented in a property mapping routine for finite element models. Additionally, a framework for stochastic finite element analysis was set up for the consideration of scatter in material properties. For validation, thin-walled parts on sub-component level were fabricated and tested in quasi-static three-point bending experiments. Elastic parameters showed considerable anisotropy, thickness dependency and scatter. A comparison of the predicted forces with experimentally evaluated reaction forces disclosed substantially improved accuracy when utilizing the novel inhomogeneous approach instead of conventional homogeneous approaches. Furthermore, the variability observed in the structural response of loaded parts could be reproduced by the stochastic simulations.

## 1. Introduction

Wherever intricate lightweight structures are sought after, production via additive manufacturing (AM) appears as promising solution, owing its capability to directly fabricate components from three-dimensional computer models in unprecedented geometrical complexity [1]. The diminished manufacturing constraints enable engineers to realize optimization-driven designs with ideal material distribution for applications in the aerospace [2,3] and automotive industries [4,5]. Large-scale structural components can be fabricated via powder bed fusion (PBF) technologies like selective laser melting (SLM) or electron beam melting (EBM) for metals as well as laser sintering (LS) for polymers and polymer matrix composites. This is on the one hand related to their good mechanical properties and on the other to the fact that these processes simultaneously offer the capability to produce relatively large parts ($10^{2}$ mm scale) at high geometrical resolution ($10^{-1}$ mm scale), the latter being pivotal for the realization lightweight designs featuring thin-walled shell structures [6].

**Figure 1:**
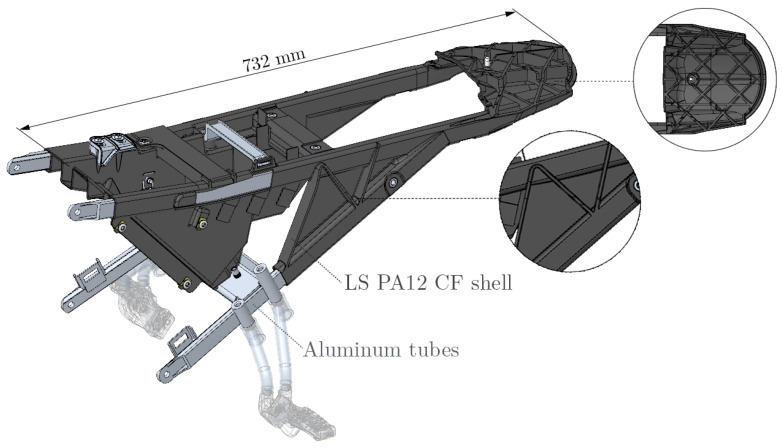
Rendering of an off-road motorcycle rear frame consisting of aluminum tubes and a LS PA12 CF structure featuring shells in various orientations and wall thicknesses. Reproduced with permission from KTM E-TECHNOLOGIES GmbH, 2021.

Besides the geometrical freedom of design, another benefit of AM lies within its potential to accelerate product development processes by rapid manufacturing of functional prototypes without the time- and resource-intensive step of tooling for validation of design iterations. If load bearing plastic components are required, the utilization of laser-sintered short-carbon-fiber-reinforced polyamide 12 (LS PA12 CF) stands to reason, as it offers significantly improved stiffness and strength compared to the plain counterpart. An example of such a full scale rapid prototype consisting of thin rib-reinforced shells for in-field design validation is shown in Figure 1.

The premise to exploit the potential of AM to reduce lead times is the accurate structural response prediction prior to part production. Thereby, the peculiar material behavior of additively manufactured structures still constitutes a major challenge. If not considered, this leads to significant deviations between numerical simulation and the actual performance observed during experimental testing [7].

A well-known characteristic of parts fabricated via PBF technologies is the anisotropy in mechanical material properties, which has been thoroughly investigated and is generally attributed to process-induced microstructures [8,9]. Typically, a considerable difference is observed between the mechanical response in build and transversal directions, which is evoked by the layer-wise manufacturing process. Within the layer direction, variations may arise as consequence of factors like the utilized infill patterns [10,11]. While these have a significant effect in other AM processes like fused deposition modeling [12], the influence of the laser hatch direction in PBF was disclosed by several authors to be minor. This indicates good agreement with transversely isotropic material laws [13,14,15,16]. This is not the case for LS PA12 CF, which exhibits a pronounced orthotropic behavior characterized by three material axes of symmetry. Like in injection molding, where the fibers align themselves with the direction of melt flow [17], in LS, the orientation occurs planar and in the direction the powder is spread over the build platform [18,19,20,21]. For each successive powder layer this spreading direction is the same, leading to alignment of fibers throughout the entire part. Therefore, additionally to the previously differentiated intra- and inter-layer behavior, PBF polymer–matrix composites exhibit a third distinct material direction, where the reinforcing function of the aligned high modulus and high strength short-fibers stands out.

The described directional behavior of PBF structures is furthermore superimposed by a severe but often overlooked thickness dependency, whereby Young’s modulus, ultimate strength and failure strain are positively correlated to part thickness. Due to the relevancy for lightweight design that relies on the utilization of thin structural members, this effect has recently gained more attention and was reported in a multitude of studies for metal- [22,23,24,25,26,27,28] as well as polymer-based PBF [29,30,31] and other processes like fused deposition modeling [32] and material jetting [33]. It appears plausible that the behavior is likewise inherent in laser-sintered short-fiber-reinforced polymers; however, no published articles were found addressing this issue. In an early study that investigated the thickness dependency of LS PA12, no decline of mechanical performance with decreasing feature size was disclosed [34]. However, the present state of knowledge suggests that the evaluated thicknesses were above the threshold below which the transition between bulk and thin-walled mechanical response occurs. The mechanisms underlying this phenomenon are not fully elucidated, but as dominant factors, microstructural variations between core and boundary areas, arising from different energy input of hatch and contour laser scan paths, as well as surface roughness that decreases the effective load bearing cross-section, are stated.

Research investigating the combined influence of build orientation and part thickness on mechanical properties by example of LS PA12 disclosed that the degree of anisotropy increases as structures become thinner [31]. Consequently, the standard options offered by current commercial finite element (FE) analysis programs to define anisotropic material constants is insufficient to predict the structural response of thin-walled additively manufactured structures [7] that are governed by inhomogeneous material properties depending on the local part orientation and thickness.

Implementation of advanced multi-scale simulation approaches that capture the internal material micro-structure based on homogenization of representative volume elements (RVE) yields anisotropic material models that reflect characteristics like distribution and orientation of reinforcement fibers [35]. However, this approach is only efficient if a small number of RVEs needs to be homogenized. Assuming that the thickness effect is strongly dependent on the surface morphology of a part, the thickness dependency of elastic parameters could only be depicted by a multitude of RVEs, differentiating between core and boundary regions and subsequent mapping throughout the component model. If feasible at all, it would be a highly laborious, complex and computationally expensive task. Another approach that could potentially depict the degraded mechanical performance of thin structures would be detailed finite element discretization of the surface morphology based on X-ray micro-computed tomography (XCT) data, as was shown on small coupon sections [36]. However, the vast efforts to acquire the three-dimensional surface characteristics via XCT and representation thereof by means of finite element meshing hinder the applicability for large-scale components in an industrial context.

To overcome these issues, in a preceding study of the author’s research group, a novel strategy was proposed to automatically map transversely isotropic and thickness-dependent material properties into finite shell element models [37]. Comparisons between numerical simulations and physical experiments on a laser-sintered thin-walled sub-component disclosed significantly improved accuracy when inhomogeneous instead of homogeneous anisotropic material properties were implemented. Thereby, the improvement is achieved by automatically creating and assigning an individual material property for each combination of shell element thickness and orientation in the entire model. Depending on the size of the FE mesh, this process takes a long time, and the exorbitant number of material property definitions complicates the handling of the model—both factors mitigating the applicability of the method in an industrial setting. Another limitation is the approach’s capability to solely depict materials with isotropic behavior in transverse direction, making it not applicable for the simulation of structures exhibiting fully orthotropic behavior like laser-sintered polymer matrix composites.

Hence, the aim of this research is to develop a new efficient linear elastic material modeling and property mapping approach that is suited for structural response prediction of inhomogeneous thickness-dependent, orthotropic shell structures, by example of LS PA12 CF.

- The first objective is the quantification of the combined effects of part thickness and build orientation on the elastic material parameters Young’s modulus and Poisson’s ratio, which, to the author’s knowledge, have not yet been disclosed for the considered material in scientific literature.
- The second objective is the derivation of a novel material modeling approach describing the thickness-dependent, orthotropic behavior based on the experimental data.
- The third objective is to improve efficiency by implementing a clustering algorithm for the mapping of the inhomogeneous material response in order to reduce the number of defined properties in the FE model.
- The last objective is the validation of the proposed modeling approach by comparing numerical simulation results with physical experiments on fabricated thin-walled parts. Thereby, not only a deterministic FE analysis building upon mean values of the tensile tests will be considered but also a stochastic framework that is intended to depict the structural response affected by scatter of the material properties.

## 2. Materials and Methods

### 2.1. Sample Fabrication

In this research, one build job comprising tensile coupons as well as thin-walled ribbed parts were produced of carbon-fiber-reinforced polyamide 12 (PDX, Protodynamix GmbH, Wetzikon, CHE) at one build height on a commercial LS system (sPro 140, 3D Systems, Inc., Rock Hill, SC, USA). It features a single 70 W CO${}_{2}$ laser and uses a scan strategy involving a contour as well as hatch scans that alternate in the two principal directions of the build platform (*x* and *y*). The powder layer thickness was 0.1 mm.

**Figure 2:**
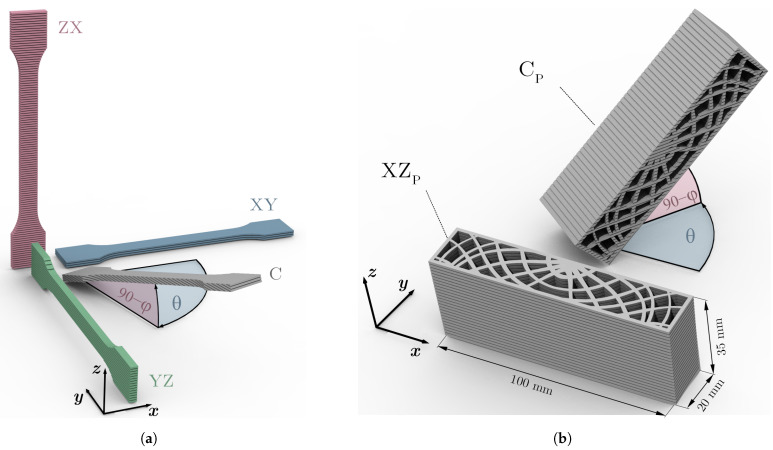
Visualization of specimen orientations with (**a**) tensile coupons for material model setup (XY, ZX, XZ) and control thereof (C) as well as (**b**) a thin-walled part of variable thickness in principal axes aligned (XZ${}_{P}$) and arbitrary (C${}_{P}$) orientations.

The tensile coupons designated for determination of the elastic material parameters were produced in three thicknesses and orientations, with dimensions according to ISO 527 [38]. As specified in ISO 527, a thickness of 4 mm was defined for the standard dogbone-shaped tensile coupons (type 1A). To represent thin-walled structures, specimens of geometry ISO 527 type 4 in thicknesses of 2 and 1 mm were chosen. The latter corresponds to the minimum achievable feature size by the system. In Figure 2a, the coupon orientations are visualized by example of the type 1A specimens.

The three variants used to set up the material model, namely XY, YZ and ZX, are aligned with the material coordinate system (i.e., the global build chamber system), whereby the principal coupon axes are oriented in *x*-, *y*- and *z*-directions, respectively. In the used nomenclature, the first letter indicates the loading and the second one the in-plane transversal direction of the tensile specimens. In addition to the aforementioned specimens that are necessary to construct the material model, another variant (C) of 1.8 mm thickness and general orientation ($\varphi =70^{\circ },\theta =75^{\circ }$) was fabricated to validate the approach proposed in Section 2.3.2. Five samples were fabricated for each orientation and thickness, summing up to a total of 50 tested tensile specimens.

In order to investigate the applicability of the herein developed structural response prediction approach, a part of sub-component complexity was produced in two orientations as depicted in Figure 2b. The thin-walled sandwich beam-like structure consists of a skin that encompasses a core featuring ribs of changing thickness and spatial orientation. Their arrangement was derived using the Karamba3D [39] tool embedded in the parametric design plug-in Grasshopper, of the Rhinoceros^®^ (Robert McNeel & Associates, Seattle, WA, USA) software package. For a three-point bending load case, these ribs are oriented along the principal stress trajectories, which themselves are lines tangent to the principal stress directions. This principle for the efficient layout of structural members was for lightweight design purposes fundamentally described by Michell [40] and has in context of AM recently re-gained attention [41,42]. The outer part dimensions of 20 × 35 × 100 mm${}^{3}$ were chosen in compliance with previous related research [43]. The two fabricated orientations comprised a part oriented with its first two principal axes parallel to the global xz-directions (XZ${}_{P}$) and another generally oriented variant ($\varphi =50^{\circ },\theta =55^{\circ }$) to test the accuracy of the simulation routine for structures deviating from the build chamber axes (C${}_{P}$). After completion of the build job, the excess powder was removed by means of blasting with glass beads. All parts were tested in as-built condition.

### 2.2. Experimental Testing

**Figure 3:**
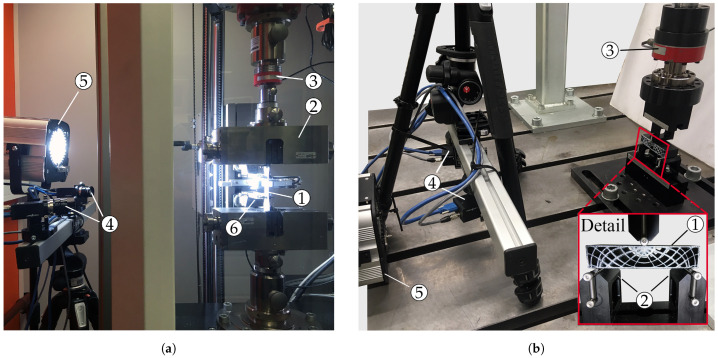
Experimental setups for (**a**) tensile and (**b**) bending tests including DIC system with respective specimen ①, fixture ②, load cell ③, DIC cameras ④, spotlight ⑤ and extensometer ⑥.

In order to obtain the elastic material parameters, uni-axial tensile tests in accordance with standard ISO 527 [38] were performed, as shown in Figure 3a, on a ZwickRoell Z100 testing machine (ZwickRoell GmbH & Co. KG, Ulm, GER), featuring a 10 kN load cell and a contact extensometer for determination of load–displacement data. The quasi-static test rate was set to 5 mm/min. Testing the coupons in the XY, YZ and ZX build orientations (see Figure 2a) allowed the determination of Young’s moduli $E_{x}$, $E_{y}$ and $E_{z}$ as well as Poisson’s ratios $\nu_{xy}$, $\nu_{yz}$ and $\nu_{zx}$, respectively. For the evaluation of the latter, a contact-less stereo digital image correlation (DIC) system was used. Based on the deformation of an air brushed speckle pattern applied on the coupon surface, it allows the computation of the strains in loading ($\varepsilon_{L}$) and transversal directions ($\varepsilon_{T}$) [9,37,44]. Consequently, $\nu_{\mathrm{LT}}=-\varepsilon_{T}/\varepsilon_{L}$ [9] yields the Poisson’s ratio for the respective coupon orientation. The DIC setup consisted of two 5 MP cameras (Grasshopper3, FLIR Integrated Imaging Solutions, Inc., Wilsonsville, OR, USA) mounted on a tripod (see Figure 3a). The captured images were post-processed in the software Vic-3D 9 (Correlated Solutions Inc., Irmo, SC, USA).

Figure 3b shows the quasi-static test setup of the thin-walled ribbed part that was loaded in a three-point bending configuration. The procedure involved a 5 N pre-load to ensure contact between supports and test specimen, followed by a 5 mm/min ramp until fracture of the beam. This rate was chosen to match the one employed in the tensile tests. For loading, a 10 kN servo-hydraulic cylinder (ZwickRoell LH 10) featuring a 10 kN load cell (ZwickRoell DR-F) was utilized. The indenter and supports of the bend fixture (MTS 642.01A-02, MTS Systems Corp., Eden Prairie, MN, USA) were cylindrical rollers with a diameter of 5 mm. The bearings were spaced 80 mm apart, and test rate was set equivalent to the one employed in the tensile tests. All samples as well as the rollers were marked to ensure accurate and repeatable positioning of the specimens. Like for the tensile coupons, a speckle pattern was air brushed onto the surface of the samples to enable contact-less evaluation of the deformation behavior during the test.

### 2.3. Numerical Simulation

Within the scope of this study a deterministic FE analysis, wherein material parameters are defined as scalar values as well as a stochastic FE analysis that yields an output response affected by scatter by defining elastic parameters including their probabilistic distribution, were considered. For both approaches the geometry discretization and boundary conditions were the same.

#### 2.3.1. Finite Element Mesh and Constraints

**Figure 4:**
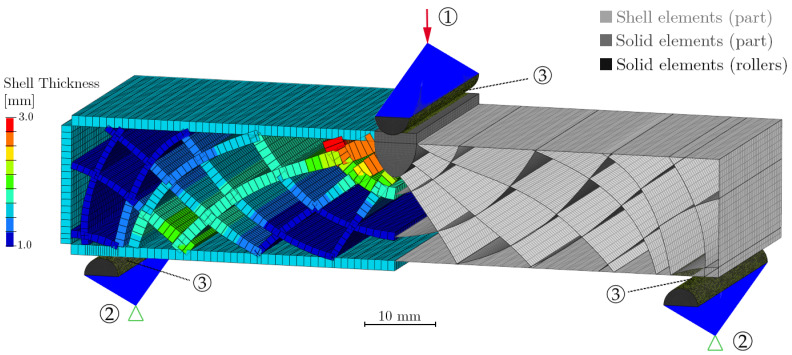
FE model in 2D shell representation (**right**), with indicated element thickness (**left**), multi-point constraints for load introduction ① and supports ② as well as contact surfaces ③. For reference to colors, please refer to the online version of this article.

The CAD geometries of the bending beams were discretized using the software HyperMesh^®^ 2020 (Altair Engineering, Inc., Troy, MI, USA). For the thin-walled ribs, a mid-surface model was generated and meshed with first-order 2D quadrilateral elements (Quad4), as depicted in Figure 4 on the right. To allow connection of shells with the core and the surrounding skin, in the latter, an offset of −(t/2) was implemented.

The shell thickness information, as visualized in Figure 4 on the left, was automatically extracted from the solid geometry and assigned to each element. As is elaborated in Section 2.3.2, this practical functionality plays an integral role for the material property mapping strategy.

At the top center, several ribs merge and form a bulky region, which was represented via solid hexahedrons (Hex8). The solid and shell meshes share the nodes at the connecting edge. For the numerical simulation, not only the part itself but also the load introducing and supporting rollers were meshed. These were modeled as half cylinders using hexahedral elements (see Figure 4).

The boundary conditions were set up using 1D multi-point constraints (RBE2). The supporting rollers were fixed in all degrees of freedom and as load, an enforced displacement of 1.5 mm was applied perpendicularly to the part’s top face in 20 incremental steps. The displacement value was selected in accordance with the outcomes of the preceding experimental bending tests. Between rollers and part, sliding contacts were defined. The bending fixture design featured polished steel rollers, which is why frictional forces were assumed to be negligibly small.

All simulations were set up as static geometric non-linear analyses and the models computed employing the Optistruct™ solver. Displacement and reaction force at the independent node of the load introducing multi-point constraint as well as the first principal strain were defined as output.

The shell element size was 1 mm, which was chosen based on a mesh convergence study. Therein, the element size was decreased until a termination criterion of 0.2% change in the response (i.e., reaction force during loading) was reached. For improved contact convergence in the non-linear analysis, the mesh constituting the contact surfaces was further refined to an element size of 0.25 mm. This led to a total number of 52,150 elements in the FE model (2D and 3D combined). This is true for both, XZ${}_{P}$ and C${}_{P}$ variants, for which only the reference coordinate system was changed.

The remaining step before being able to run a simulation is the definition of material properties. For the rollers, standard isotropic steel properties were assigned. The definition of the inhomogeneous LS PA12 CF material behavior is elaborated in-depth in the next section.

#### 2.3.2. Material Modeling

**Figure 5:**
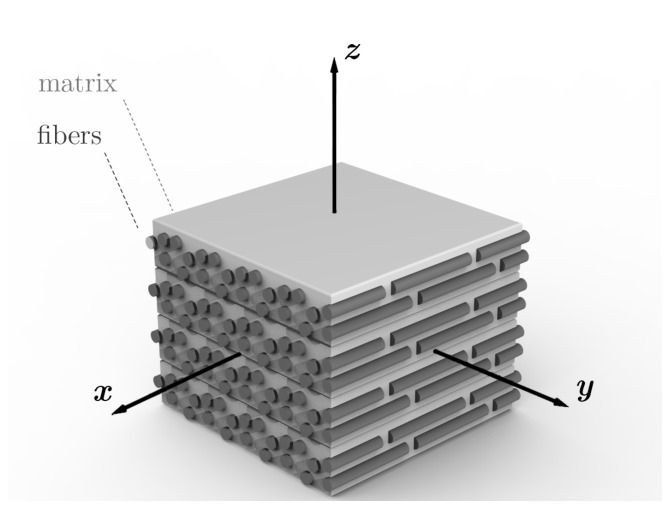
Rendering of an RVE with aligned fibers, powder spreading (*x*), transversal (*y*) and build directions (*z*).

Considering the aforementioned findings that in LS PA12 CF there are three axes of material symmetry that coincide with the global build coordinate system (see Section 1), in Figure 5, a representative volume element (RVE) is visualized. The sintering layers of the matrix material, displayed in light gray, are oriented in the xy-plane and are stacked in build direction (*z*). The reinforcement fibers are aligned in *x*-direction. Accounting for the symmetry conditions in Equation (1a–c), the compliance matrix $S^{H}$ for such an homogeneous orthotropic material depends on nine parameters and can be written as given by Equation (1d) [45].

(1a) $$\frac{\nu_{xy}}{E_{x}}=\frac{\nu_{yx}}{E_{y}},$$

(1b) $$\frac{\nu_{zx}}{E_{z}}=\frac{\nu_{xz}}{E_{x}},$$

(1c) $$\frac{\nu_{yz}}{E_{y}}=\frac{\nu_{zy}}{E_{z}},$$

(1d) $$S^{H}=\left[\begin{array}{cccccc} \frac{1}{E_{x}} & \frac{-\nu_{xy}}{E_{x}} & \frac{-\nu_{zx}}{E_{z}} & 0 & 0 & 0\\ \; & \frac{1}{E_{y}} & \frac{-\nu_{yz}}{E_{y}} & 0 & 0 & 0\\ \; & \; & \frac{1}{E_{z}} & 0 & 0 & 0\\ \; & \; & & \frac{1}{2G_{yz}} & 0 & 0\\ \; & \mathrm{sym}. & \; & & \frac{1}{2G_{xz}} & 0\\ \; & \; & & \; & & \frac{1}{2G_{xy}} \end{array}\right]$$

comprising Young’s moduli $E_{i}$, Poisson’s ratios $\nu_{ij}$ and shear moduli $G_{ij}$, where indices denote the global coordinate axes in the sequence of cause and effect. Using a notation including $\nu_{zx}$ instead of $\nu_{xz}$ allowed the direct determination of Young’s moduli and Poisson’s ratios required to populate the matrix based on tensile testing of three coupon orientations (XY, YZ and ZX) only. Shear moduli, on the other hand, were approximated by Equation (2)

(2) $$G_{ij}=\frac{\sqrt{E_{i}E_{j}}}{2(1+\sqrt{\nu_{ij}\nu_{ji}})}$$

which was proposed by Huber [46]. Recent publications yielded satisfactory results using Equation (2) for LS materials [9,37,47]. The remaining Poisson’s ratios that were not directly determined in the experiments were computed using Equation (1a–c).

This typical formulation describes the orthotropy without thickness dependency that is readily available in FE software tools, which, on the present platform, is represented by the MAT9ORT material card. For the central bulk region of the ribbed parts that were discretized using solid elements, such behavior was defined using the experimentally derived elastic constants of the thick (*t* = 4 mm) tensile coupons and shear moduli derived by Equation (2). To account for the different build orientations of the parts XZ${}_{P}$ and C${}_{P}$, local material coordinate systems were assigned.

In contrast to the solid region, for the thin-walled ribbed sub-structure (t= 1–3 mm) thickness dependency was considered. In a preceding work, the thickness effect of Young’s modulus and Poisson’s ratio for transversely isotropic LS PA12 without reinforcement fibers was effectively modeled by fitting of Weibull growth and a two terms exponential functions to experimental data, respectively [37]. A similar empirical approach was pursued in the present research, where instead of the previous two models, a single three parameter model was selected. By non-linear least squares fitting (Curve Fitting Toolbox™, The MathWorks, Inc., Natick, MA, USA), the three-parameter model constitutes a function that interpolates mean Young’s modulus and Poisson’s ratio data as a function of thickness. The power law utilized to model the thickness dependency for the three coupon orientations XY, YZ, ZX is given in Equation (3) [48]

(3) $$f\left(t\right)=a\;t^{b}+c\;\mathrm{for}\;t\geq t_{\min}$$

with the variable shell thickness *t*, as well as the respective fitted model coefficients *a*, *b* and *c*, representing the scaling factor, exponent and deviation term, respectively. Thereby, the lower limit of *t* is the minimum achievable feature size $t_{\min}$ by the used manufacturing system.

Fitting a function according to Equation (3) to the data determined via the three coupon orientations yields the predicted thickness-dependent Young’s moduli ($E_{x}\left(t\right)$, $E_{y}\left(t\right)$ and $E_{z}\left(t\right)$) as well as Poisson’s ratios ($\nu_{xy}\left(t\right)$, $\nu_{yz}\left(t\right)$ and $\nu_{zx}\left(t\right)$) for an element that is aligned with the material coordinate system. Substituting the parameters given in Equation (2) by these thickness-dependent functions approximates the respective shear moduli ($G_{xy}\left(t\right)$, $G_{yz}\left(t\right)$, $G_{xz}\left(t\right)$). Utilizing the nine thickness-dependent functions instead of the engineering constants shown in Equation (1d) yields the orthotropic compliance matrix $S^{I}\left(t\right)$ specifically for a structure of inhomogeneous thickness *t*, as given in Equation (4)

(4) $$S^{I}\left(t\right)=\left[\begin{array}{cccccc} \frac{1}{E_{x}\left(t\right)} & \frac{-\nu_{xy}\left(t\right)}{E_{x(t)}} & \frac{-\nu_{zx}\left(t\right)}{E_{z}\left(t\right)} & 0 & 0 & 0\\ \; & \frac{1}{E_{y}\left(t\right)} & \frac{-\nu_{yz}\left(t\right)}{E_{y}\left(t\right)} & 0 & 0 & 0\\ \; & \; & \frac{1}{E_{z}\left(t\right)} & 0 & 0 & 0\\ \; & \; & & \frac{1}{2G_{yz}\left(t\right)} & 0 & 0\\ \; & \mathrm{sym}. & \; & & \frac{1}{2G_{xz}\left(t\right)} & 0\\ \; & \; & & \; & & \frac{1}{2G_{xy}\left(t\right)} \end{array}\right]$$

For solid meshes of a thin-walled part displaying thickness-dependent, orthotropic material behavior, utilization of $S^{I}\left(t\right)$ would be sufficient since in solid elements the constituents entered in principal material directions are typically transformed automatically if a local coordinate system indicating the material orientation is defined. However, automatic determination of local wall-thickness in a solid mesh of a complex structure is not a trivial task and is not a concern of the present research, where specifically thin-walled shell structures are in focus.

**Figure 6:**
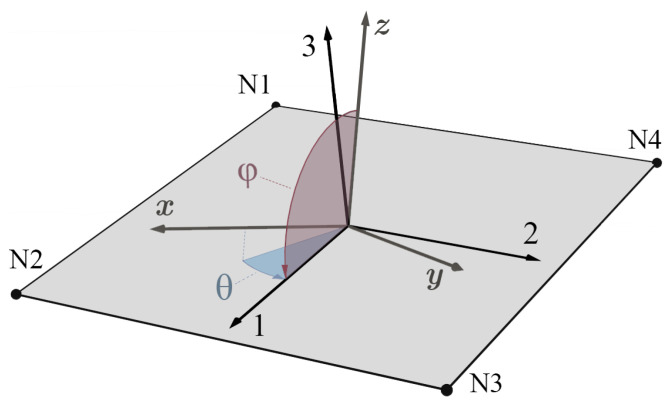
Shell element defined by nodes N1–N4 with local 123-system and azimuth θ as well as polar angle φ of the 1-direction with respect to the xyz-system.

In contrast, shell elements have the thickness information, but the local material system can in commercial FE tools merely be rotated in the element plane by definition of an azimuth angle θ. This is sufficient for carbon-fiber-reinforced plastic laminates where the fibers of the plies generally lie in the shell plane. In PBF, the material orientation is determined by the global build coordinate system (xyz) and does not follow the part geometry, which implies that the local 123-system of the shell element deviates for all but the single instance where the respective xyz- and 123-axes are parallel. In Figure 6, an arbitrarily oriented shell element is depicted. By default, its 1-direction is defined parallel to the edge from first (N1) to second node (N2) and the 2-direction as perpendicular to both the 1- and 3-directions (element normal). In orthotropic shell elements, the material behavior is determined by the so-called reduced stiffness matrix $Q^{-1}$ [49,50] that allows for the definition of material parameters corresponding to the local 1- and 2-directions. Consequently, to obtain the desired shell response, the four thickness-dependent constituents $E_{1}\left(t,\theta ,\varphi \right)$, $E_{2}\left(t,\theta ,\varphi \right)$, $\nu_{12}\left(t,\theta ,\varphi \right)$ and $G_{12}\left(t,\theta ,\varphi \right)$ of the local reduced stiffness matrix $Q^{-1}\left(t,\theta ,\varphi \right)$ given in Equation (5) are computed based on $S^{I}\left(t\right)$.

(5) $$Q^{-1}\left(t,\theta ,\varphi \right)=\left[\begin{array}{ccc} \frac{1}{E_{1}\left(t,\theta ,\varphi \right)} & \frac{-\nu_{12}\left(t\right)}{E_{1}\left(t,\theta ,\varphi \right)} & 0\\ \; & \frac{1}{E_{2}\left(t,\theta ,\varphi \right)} & 0\\ \mathrm{sym}. & \; & \frac{1}{G_{12}\left(t,\theta ,\varphi \right)} \end{array}\right]$$

Based on an approach detailed by Nordmann, Aßmus and Altenbach [45], the Young’s modulus for an arbitrary direction in three-dimensional space can be derived of $S^{I}\left(t\right)$, which for the shell element modulus in 1-direction $E_{1}\left(t,\theta ,\varphi \right)$ yields Equation (6)

(6) $$E_{1}\left(t,\theta ,\varphi \right)={\left[\begin{array}{c} d_{V1}^{⊤}\;S^{I}\left(t\right)\;d_{V1} \end{array}\right]}^{-1}\;\mathrm{with}\;d_{V1}=\left[\begin{array}{c} d_{1_{1}}d_{1_{1}}\\ d_{1_{2}}d_{1_{2}}\\ d_{1_{3}}d_{1_{3}}\\ \sqrt{2}d_{1_{2}}d_{1_{3}}\\ \sqrt{2}d_{1_{1}}d_{1_{3}}\\ \sqrt{2}d_{1_{1}}d_{1_{2}} \end{array}\right]\;\mathrm{and}\;d_{1}=\left[\begin{array}{c} \sin(\varphi )\cos(\theta )\\ \sin(\varphi )\sin(\theta )\\ \cos(\theta ) \end{array}\right]$$

involving for the present study, the compliance matrix derived using thickness-dependent parameters $S^{I}\left(t\right)$ and the dyadic product of the direction vector $d_{1}$ in Voigt [51] notation $d_{V1}$. For determination of the shell element’s second Young’s modulus $E_{2}\left(t,\theta ,\varphi \right)$, the vector in 2-direction $d_{2}$ is derived based on its corresponding φ and θ angles, of which $d_{V2}$ can be computed analogously to $d_{V1}$. Poisson’s ratio and shear modulus necessitate the consideration of the transverse direction. To obtain the two shell element material parameters, consequently, Equation (7)

(7) $$\nu_{12}\left(t,\theta ,\varphi \right)=E_{1}\left(t,\theta ,\varphi \right)\;d_{V1}^{⊤}\;S^{I}\left(t\right)\;d_{V2}$$

and Equation (8)

(8) $$G_{12}\left(t,\theta ,\varphi \right)={\left[\begin{array}{c} 2m_{V}^{⊤}\;S^{I}\left(t\right)\;m_{V} \end{array}\right]}^{-1}\;\mathrm{with}\;m_{V}=\frac{\sqrt{2}}{2}\left[\begin{array}{c} 2d_{1_{1}}d_{2_{1}}\\ 2d_{1_{2}}d_{2_{2}}\\ 2d_{1_{3}}d_{2_{3}}\\ \sqrt{2}\left(d_{1_{2}}d_{2_{3}}+d_{2_{2}}d_{1_{3}}\right)\\ \sqrt{2}\left(d_{1_{1}}d_{2_{3}}+d_{2_{1}}d_{1_{3}}\right)\\ \sqrt{2}\left(d_{1_{1}}d_{2_{2}}+d_{2_{1}}d_{1_{2}}\right) \end{array}\right]$$

were employed that, analogous to $d_{V1}$ in Equation (6), depend on $d_{V2}$ and $m_{V}$, respectively. Regarding further details, the reader is referred to Nordmann et al. [45], who focused on the derivation of these fundamental equations in their article.

Using the described methodology, for each unique combination of shell element thickness and orientation in the FE mesh, material parameters are computed that, in the case of a fully mapped model, are used to generate and assign a corresponding number of different material properties. This implies that in the fully mapped case, the number of property definitions in the FE model is equal to the number of unique element configurations.

#### 2.3.3. Property Clustering

One objective of this study was to reduce the number of created material properties in the model, while maintaining the inhomogeneous material behavior for the structural response prediction. Therefore, a *k*-means++ clustering algorithm [52] was integrated within the mapping script due to its fast convergence and straight forward implementation compared to other partitioning methods [53]. The basic idea behind this iterative algorithm is to partition a data set of *n* sample points in a pre-defined number of clusters *k*, so that the sum of the squared Euclidean distances *J* from each multidimensional data point $x_{i}$ to its corresponding cluster center $\mu_{j}$ is minimized, i.e., Equation (9) [53]

(9) $$J=\sum_{j=1}^{k}\sum_{i=1}^{n}\left|\right|x_{i}-\mu_{j}{\left|\right|}^{2}\;\to \;\min\;\mathrm{with}\;\mu_{j}=\frac{1}{n}\sum_{i=1}^{n}x_{i}$$

Since grouping of the elements based on thickness and orientation angles is, due to the non-linear relationship with the material parameters, not sensible, the apparent material properties were calculated for each element ($x_{i}$) and clustered thereafter. Consequently, each sample point in the data set has four dimensions, which are namely $E_{1}\left(t,\theta ,\varphi \right)$, $E_{2}\left(t,\theta ,\varphi \right)$, $\nu_{12}\left(t,\theta ,\varphi \right)$ and $G_{12}\left(t,\theta ,\varphi \right)$. To assort equal weight to all of the dimensions of unequal units, normalization was performed prior to partitioning using the *z*-score method [54].

After convergence of the algorithm, the obtained cluster centroids $\mu_{k}$ are assigned to the corresponding elements in the FE model, reducing the number of defined material properties from *n* to *k*. While an arbitrary value between 1≤k≤n can be chosen, within the present methodology, in which a high degree of automation was aspired, the elbow method was employed for automated determination of an efficient number of clusters for the considered part. Thereby the squared distance criterion *J* is evaluated and plotted over a range of *k* values. As the number of clusters increases, the total within cluster variation in the data set decreases, typically depicting an “elbow-shaped” graph. Automated determination of the change points of this curve [55] yields an optimal *k*-value so that adding another cluster does not substantially reduce the total within cluster variation [53]. For the setup of the deterministic FE models, both fully mapped and clustered material properties were considered.

#### 2.3.4. Stochastic Property Sampling

To account for the inherent scatter in mechanical material properties, a stochastic FE analysis was set up. In contrast to deterministic FE models, where the material parameters are represented by single (mean) values, stochastic approaches additionally allow the implementation of property variability by defining statistical distributions based on mean and standard deviations. Consequently, solving models with variable material properties generated by random sampling methods yields scatter in the predicted structural response [56].

As previously mentioned, the number of property definitions in a fully mapped model can be very large, as it equals the number of unique combinations of shell element thickness and orientation in the entire FE mesh. Since in the considered structure the number of unique properties is close to 300 (see Section 3.4.1) and each consists of four material parameters, random sampling of 300 × 4 features would lead to an amount of FE models that cannot be solved in any justifiable time frame. Consequently, in the present study, the 5 clustered material properties employed in the deterministic analysis were used as nominal mean values in the stochastic approach. The hypothesis of normally distributed tensile test sample data could, based on the statistical Anderson Darling test with a significance level of 5%, not be rejected and complies with previous findings regarding mechanical properties of LS PA12 CF [20]. For the definition of the normal distribution, besides the mean value, the standard deviation is necessary. To obtain the corresponding standard deviations *s* as functions of thickness and orientation, the same approach as that described in Section 2.3.2 was repeated, but instead of fitting the model given in Equation (3) to the mean values $\bar{x}$ of the experimental data, it was fitted through the upper ($\bar{x}+s$) and lower bounds ($\bar{x}-s$), respectively. This enabled the compilation of the upper and lower bounds of the thickness-dependent compliance matrices $S^{\mathrm{upper}}\left(t\right)$ and $S^{\mathrm{lower}}\left(t\right)$ from which the material property variation for elements of any thickness and orientation is extrapolated. Consequently, the mean standard deviation was obtained by taking the square root of the averaged variances of the properties assigned to the respective clusters.

The next step included the variation of the random variables according to the formulated distributions. Therefore, a Latin hypercube procedure [57] incorporated in the Hyperstudy^®^ software was used to generate 100 samples. The number of samples is positively correlated with the reliability of the generated output and is limited by the time to solve a multitude of models depicting geometric and contact non-linearity. The chosen number of samples consequently represents the maximum justifiable number of runs within the scope of this study and, hence, must be seen as trade-off between reliability and necessary time to solve all variations. Apart from the considered scatter in material behavior, the stochastic FE model was set up identical to the deterministic one, with boundary conditions as described in Section 2.3.1.

## 3. Results

### 3.1. Elastic Material Parameters

Table 1 comprises the experimentally determined Young’s moduli and Poisson’s ratios of principal orientations and thicknesses, designated for definition of the material model. For Young’s modulus, minimum and maximum values amounted to 2704 and 6920 N/mm${}^{2}$ and were obtained for the 1 mm *z*- and 4 mm *x*-directions, respectively. The lowest Poisson’s ratio of 0.210 was observed in the 1 mm ZX coupons, while the highest value of 0.488 was scored for the 4 mm YZ coupon orientation. The magnitude of the material parameters for the different orientations as determined by the 4 mm thick tensile coupons lies within the range reported in related research investigating LS PA12 CF [19,20]. Regarding thin specimens with wall-thickness t≤2 mm, no published data were found for the mechanical properties of this material.

The material parameters exhibited orthotropic behavior, whereby in both cases, one orientation deviated more drastically from the others, which is related to the aforementioned preferred alignment of carbon fibers in *x*-direction. The directional reinforcement effect of the polymer matrix composite on the one hand leads to considerably elevated performance in $E_{x}$ and on the other hand to lower values in $\nu_{zx}$, where transverse contraction is hindered [50].

**Figure 7:**
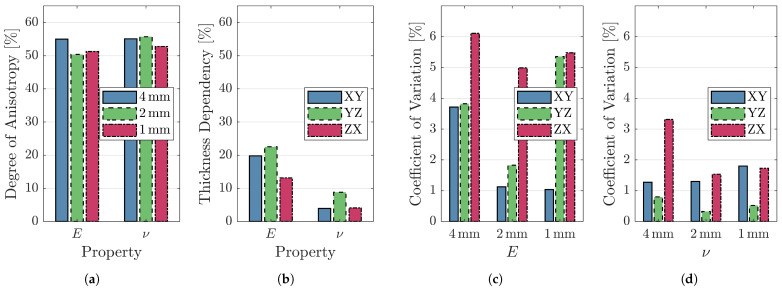
Maximum relative differences indicating (**a**) degree of anisotropy for each thickness and (**b**) thickness dependency for each orientation as well as scatter in (**c**) Young’s modulus *E* and (**d**) Poisson’s ratio ν.

As depicted in Figure 7a, the degree of anisotropy, calculated as the relative difference between the highest and lowest scoring build orientations, is similar for all coupon thicknesses and levels around 50% for both Young’s modulus and Poisson’s ratio.

In contrast to pure laser-sintered PA12, where the thickness dependency exerted a greater effect on the mechanical properties than anisotropy [31], the present material shows a less sensitive response. Nevertheless, a significant maximum relative difference exceeding 20% between coupons of 1 and 4 mm wall-thickness prevailed (see Figure 7b).

Figure 7c reveals that for all thicknesses, the XY orientation showed the lowest and ZY the highest coefficient of variation in Young’s modulus. Poisson’s ratio showed the lowest scatter in YZ oriented coupons across thicknesses (see Figure 7d). The highest coefficient of variation was apparent in the 4 mm thick ZX specimens. This, however, was not the case for all thicknesses.

### 3.2. Material Model

**Figure 8:**
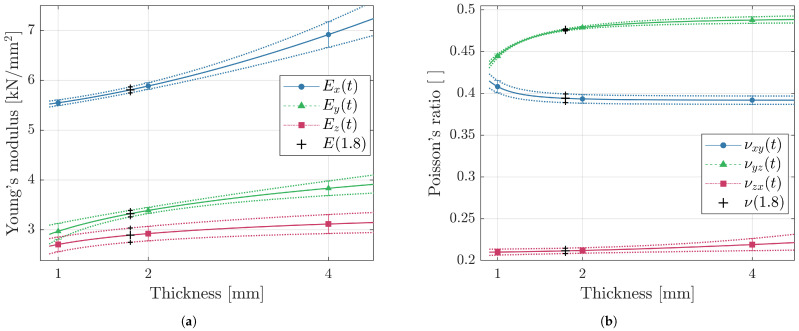
Thickness dependency for principal coupon orientations with continuous curves fitted to empiric means shown as markers as well as dotted lines fitted to the upper and lower bounds of the standard deviations indicated by the error bars for (**a**) Young’s modulus and (**b**) Poisson’s ratio.

The first step in the present approach was to model Young’s modulus and Poisson’s ratio as functions of thickness by fitting a parametric function (see Equation (3)) to experimental data. Figure 8 shows for each build orientation the fitted curves passing through experimentally mean values as well as upper and lower standard deviation bounds, which are represented as markers and error bars, respectively.

As stated in Section 2.1, an additional coupon not utilized to set up the model (see C in Figure 2a), of 1.8 mm thickness and orientation deviating from the global axes ($\varphi =70^{\circ },\theta =75^{\circ }$), was fabricated and tested. This is utilized to check the validity of the modeling approach at the coupon stage by comparing the experimentally determined and predicted material behavior. To obtain the respective prediction value, firstly the coupon thickness t=1.8 mm is inserted in the six functions shown in Figure 8, which yields the parameters necessary to approximate the remaining three shear moduli (see Equation (2)) for construction of the thickness-dependent compliance matrix $S^{I}\left(1.8\right)$. Inserting polar φ and azimuth angles θ into equations Equations (6) to (8) allows the derivation of the four thickness and orientation dependent parameters ($E_{1}\left(t,\theta ,\varphi \right)$, $E_{2}\left(t,\theta ,\varphi \right)$, $\nu_{12}\left(t,\theta ,\varphi \right)$, $G_{12}\left(t,\theta ,\varphi \right)$) necessary to formulate the elastic behavior of a shell element $Q^{-1}\left(t,\theta ,\varphi \right)$. Likewise, the upper and lower prediction bounds are approximated for the given thickness and orientation.

**Figure 9:**
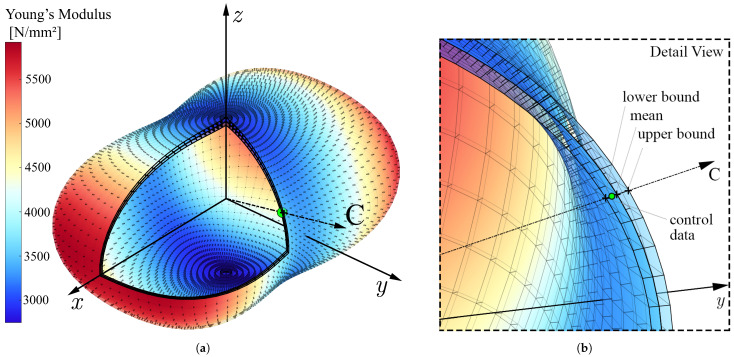
(**a**) Visualization of anisotropy in Young’s modulus for an element of t=1.8 mm, with mean, upper and lower standard deviation bounds represented by the three surface plots and (**b**) detailed view of the experimentally obtained control data point of coupon C indicated by the green marker.

In Figure 9a, the anisotropic elastic response of the 1.8 mm thick control coupon is exemplary depicted for Young’s modulus. Therein, the three nested surface plots show the predicted mean as well as upper and lower standard deviation bounds. The orientation of the control coupon is indicated by the dash dotted axis referred to as C, and the experimentally determined Young’s modulus is shown as green marker. As visible in the detailed view (see Figure 9b), the empiric mean value lies below the predicted mean but within the lower standard deviation bound. The marginal difference of 36 N/mm^2^ suggests very good agreement between experiment and model.

Based on this described routine, a script automatically calculates the thickness and orientation dependent material properties for each shell element in the model. The data are stored in a variable and can either be directly assigned to the respective element to obtain a fully mapped model or can be partitioned into clusters of similar behavior before creation and assignment of properties. The latter is elaborated in the next section.

### 3.3. Clustered Material Properties

**Figure 10:**
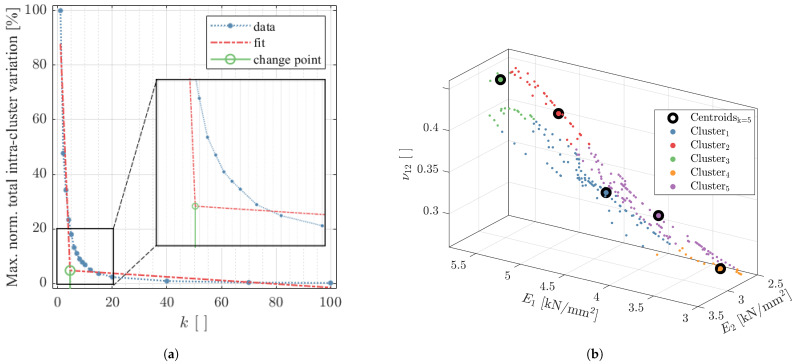
(**a**) Elbow plot with optimal number of clusters indicated in green and (**b**) visualization of material properties partitioned into five clusters with corresponding centroids for part XZ${}_{P}$.

To avoid thousands of potentially only marginally different material properties in the mapped FE model, it was an objective of the present research to implement a clustering procedure. The elbow plot method [53] to determine the optimal number of clusters used in this study is depicted in Figure 10a by example of the XZ${}_{P}$ part (see Figure 2b).

The material properties computed for the shell model were clustered for different values of *k* between 1 ≤k≤ 100, and for each, the total intra-cluster variation (i.e., total within-cluster sum of squared errors) was plotted over the respective *k*-value. Automatic derivation of the change point by finding the intersection of two best fit lines yielded the value of 4.5. Since decimal numbers of clusters cannot be defined, the next greater value (i.e., k=5) was chosen.

Figure 10b shows the apparent shell material properties calculated for XZ${}_{P}$ color coded by the assigned cluster as well as the resulting five centroids that resemble the properties that will be assigned in the FE model. The plot indicates that the Young’s modulus in the element 2-direction levels around 3000 N/mm${}^{2}$, which is related to the considered XZ orientation of the part, as in the model, the 2- is aligned with the global *z*-direction. Consequently, the 1-direction was always in the xy-plane and varied in azimuth angle depending on the trajectory of the curved ribs, leading to a wider spread between minimum and maximum values of $E_{1}$. Furthermore, the results imply that the in-element-plane Poisson’s ratio $\nu_{12}$ tended to increase with $E_{1}$. At this point, it must be noted that shear modulus was also considered as a feature in the algorithm; however, it was omitted from the visualization to allow a graph in three dimensions.

### 3.4. Structural Response Prediction

#### 3.4.1. Deterministic Analysis

**Figure 11:**
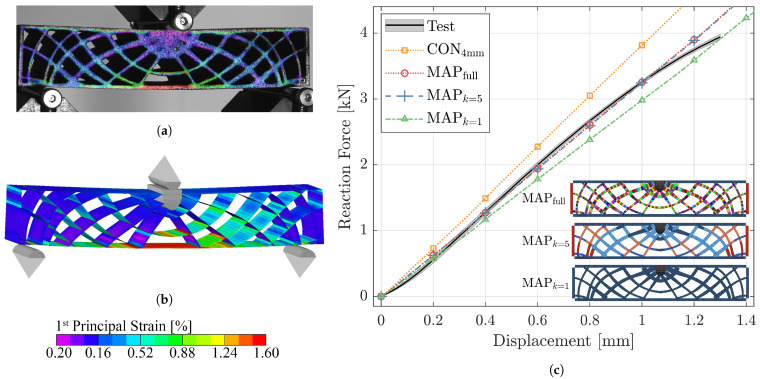
Contour plots of first principal strain at 1.3 mm displacement derived of (**a**) DIC measurements of XZ_P_ and (**b**) numerical simulation of the corresponding MAP_full_ model as well as (**c**) comparison of reaction force–displacement curves for mean experimental data with standard deviation displayed as shaded area and deterministic FE analyses, including constant, fully mapped and clustered models exemplarily for the XZ_P_ part. Color codes indicate property distribution in the mapped FE models.

In Figure 11, the results of the deterministic FE analysis for the XZ oriented part (XZ${}_{P}$) are comprised. The comparison of the first principal strains as captured by DIC (see Figure 11a) and computed for the virtual model (see Figure 11b) indicates good agreement between the physical experiment and numerical simulation. At the indenter displacement of 1.3 mm, the contour plot in both DIC and FE analysis shows a similar distribution and magnitude, with the strain maximum occurring in the center of the bottom skin, which is expected for the given load case.

Figure 11c shows the reaction force acting in vertical direction on the indenter plotted over its displacement for the experiment as well as simulations based on different material modeling and clustering approaches.

The curve denoted with Test is the average obtained from five bending beam samples with standard deviations displayed as shaded area. The narrow band indicates low variability between the samples and high repeatability of the utilized experimental setup. The mean curve exhibits a slight sigmoid progression. From the start until approximately 0.2 mm displacement, the reaction force increases progressively, followed by a degressive trend until the maximum load of 3.94 kN. The end of the curve is the last instance before the first of the beams failed.

As baseline, one FE model was set up without property mapping, where a single material was defined based on the bulk behavior obtained from the tensile coupons of 4 mm thickness. The response predicted by this constant model (CON${}_{4\mathrm{mm}}$), in which no thickness-dependent orthotropy was considered, substantially exceeded the experimentally evaluated data. At 1 mm displacement, the difference between simulation and experiment amounted to 17.4%.

If the material properties are mapped accounting for the thickness and spatial orientation of each shell element in the beam (MAP${}_{\mathrm{full}}$), the difference at 1 mm displacement is reduced to less than 1%. As seen in Figure 11c, overall, the MAP${}_{\mathrm{full}}$ model accurately predicted the structural response of the LS structure, whereby it was despite the geometrically non-linear analysis not capable of capturing the aforementioned slight sigmoidal progression observed in the bending tests. The model comprised close to 300 different material property definitions.

The elbow method (see Figure 10a) suggested for the present structure that the inhomogeneous material behavior could be efficiently represented by five properties. In fact, the load–displacement curve, resulting from solving the model in which five clustered properties were mapped (MAP${}_{k=5}$), was almost identical (maximum error <0.5%) to the fully mapped approach. To further investigate the potential of the clustering procedure, all apparent properties in the model were averaged to a single material definition by setting k=1 in the *k*-means algorithm (MAP${}_{k=1}$). The maximum difference between test and simulation occurred at 0.9 mm and amounted to −8.3%, indicating the benefits of the material modeling and clustering approach, even if just a single property is defined.

The distributions of material properties for the three presented approaches including mapping are shown color coded in bottom right corner of Figure 11c. Due to symmetry of the XZ${}_{P}$ structure with respect to the material coordinate system (i.e., build coordinate system), the left side is mirrored about the vertical medial axis. Moreover, it is noteworthy that for each model, the top and bottom as well as the lateral skins have the same thickness and orientations, respectively. Between models, the skin properties differ depending on the number of clusters.

#### 3.4.2. Stochastic Analysis

**Figure 12:**
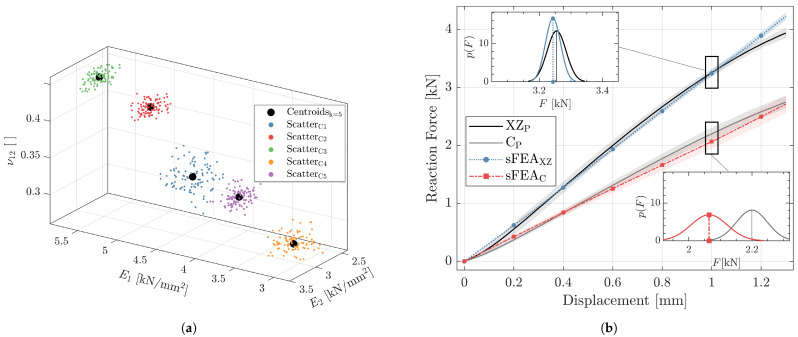
(**a**) Randomized material properties of the clustered (k=5) XZ${}_{P}$ specimen and (**b**) load–displacement curves of the stochastic analysis compared to experimental results, where shaded areas indicate the 95% confidence interval around the mean curves.

Figure 12a exemplarily shows the 100 randomized samples scattered around each of the nominal five cluster centroids, which represent the material properties defined in the MAP${}_{k=5}$ model of the XZ${}_{P}$ part. Like in Figure 10b, the fourth dimension ($G_{12}$) is omitted from the graph in order to allow plotting in three dimensions only. For each of the 100 property variations, a deterministic FE model was solved and consequently yields the average predicted structural response sFEA${}_{\mathrm{XZ}}$, which in Figure 12b is compared to the mean experimentally determined reaction force–displacement curve, including the corresponding 95% confidence interval ($\bar{x}\pm 2s$) as shaded areas. To investigate the validity of the simulation approach beyond build room coordinate system-aligned structures, the entire procedure was performed as well for the generally oriented C${}_{P}$ beam, which is indicated in the plot via the sFEA${}_{C}$ curve.

Overall, the curves indicate that the numerical simulation predicted the mean structural response for both beam orientations with high precision. The maximum deviation of the mean was 7.3% at 1.3 mm and 6.3% at 0.98 mm for the XZ and C oriented parts, respectively.

Furthermore, the predicted scatter, as indicated by the shaded area plotted around the mean curve, is in good agreement with the variability apparent in the experimental data. In the detailed views in Figure 12b, the probability density functions p(F) are plotted with vertical line indicating the mean, exemplarily for the reaction force at 1 mm displacement. One can see that the spread in p(F) deviates only slightly between experiment and prediction for both beam orientations and that the variability is higher in the off-axis oriented parts. The maximum differences in standard deviation between experiment and simulation were an underestimation of 45 N at 0.25 mm and an overestimation of 21 N at 1.3 mm for the XZ and C oriented parts, respectively.

While the relative difference between the means shown in the detailed view of Figure 12b for sFEA${}_{\mathrm{XZ}}$ and XZ${}_{P}$ lies below 1%, it is considerably larger between sFEA${}_{C}$ and C${}_{P}$, amounting to approximately 6%. This difference, however, is not constant and is due to the previously noted higher non-linearity in the experimentally determined reaction force.

## 4. Discussion

In order to improve the accuracy and reliability of structural response predictions for additively manufactured components displaying orthotropic and thickness-dependent elastic behavior affected by scatter, a material modeling approach for stochastic FE analyses was proposed. Therefore, not only were material parameters obtained in tensile tests, but ribbed parts on the sub-component level were also fabricated and experimentally loaded for validation of the numerical simulations.

Tensile tests disclosed an orthotropic material behavior characterized by an approximately 50% lower Young’s modulus and Poisson’s ratio in *z*- than in *x*-direction (see Figure 7a). This pronounced directional performance in additively manufactured polymer matrix composites is supported by previous publications on similar materials and is explained by the layer-wise build process as well as the alignment of reinforcement fibers in the powder spreading direction *x* [18,19,20,21]. The thickness dependency, which to the authors’ knowledge, has been reported for various PBF materials [22,23,24,25,26,27,28,29,30,31,33,36] but not PA12 CF, revealed in the evaluated range (1–4 mm) a reduction of up to 23 and 9% for Young’s modulus and Poisson’s ratio, respectively (see Figure 7b). This peculiar mechanical behavior in the reference articles is typically attributed to surface roughness, internal defects and micro-structural inhomogeneity. Since in the current investigation not the origins of the material characteristics but rather their implementation to FE analyses is in focus, no further investigations were carried out to confirm the aforementioned explanations. Considering the progressive increase in $E_{x}\left(t\right)$ (see Figure 8a) indicates that the transition from thickness-dependent thin to constant bulk behavior occurs later in the short-fiber-reinforced material than in plain LS PA12, where the slope leveled off towards a specimen thickness of 4 mm. Additionally different to plain LS PA12, where the degree of anisotropy was shown to be negatively correlated with wall-thickness, no obvious trend is apparent in the present reinforced material. In a broad context, this implies that the characterization of mechanical properties based on a single coupon thickness, as recommended in the ISO and ASTM test standards, is insufficient. As previously recommended by others [32], test standards for the determination of mechanical properties of additively manufactured materials should be revised to account for the characteristic inhomogeneity.

Evaluation of the scatter observed in the tensile test results disclosed maximum coefficients of variation of close to 6 and 3% for Young’s modulus and Poisson’s ratio, respectively (see Figure 7c,d). This is in good agreement with previously published data on LS PA12 CF [20].

The material modeling strategy built upon the experimentally determined effects of build orientation and wall-thickness was capable of predicting the elastic material parameters with high precision, which was approved based on the arbitrarily oriented control coupon C that is shown in Figure 9a,b. Consequently, the findings disclosed that by using the proposed property mapping method, the structural response of more complex structures displaying thickness dependency and orthotropy can be estimated at significantly improved accuracy compared to FE models with constant properties (see Figure 11). The reasons for the remaining deviations appear to derive from a slightly more non-linear progression of the reaction force measured during the experiments, despite the geometrically non-linear definition of the numerical simulation.

The progressive start of the experimentally determined XZ${}_{P}$ and C${}_{P}$ curves is likely related to the slight indentation of the load introducing and supporting rollers of the three-point bending set up. This initial plastic deformation of surface particles before the actual structure is loaded cannot be ruled out during the physical tests. In contrast, the contact interface in the discretized FE model did not account for any surface protrusions, implying direct load introduction in the beam, which would explain the more linear structural response in the simulation. This phenomenon would be influenced by the diameter of the utilized rollers, whereby larger radii would minimize localized plastic deformation of the specimen surface. Another reason for the non-linear progression apparent from 0–0.2 mm displacement during the experiments could again be related to geometrical imperfections, however, not on the microscopic level but rather regarding warpage of the entire additively manufactured beam. While the virtual FE model is perfectly flat, slight distortions in the physical part may lead to incomplete contact at low loads, which would induce a flatter slope of the force–displacement curve until complete contact is formed between specimen and supports. This effect could be diminished by increasing the pre-load during the test procedure. Inspecting XZ${}_{P}$ in Figure 11c yields that approximately 500 N, instead of the employed 5 N, would have been necessary to overcome the initial non-linearity of the reaction force. However, one has to keep in mind that, thereby, the first 15% of the displacement would have been cut off.

The degressive non-linear response observed in the experiments at larger displacements that was absent in the simulations may be related to the fact that the utilized MAT8 material cards in the FE model do not account for elasto-plastic behavior. Young’s moduli for definition of the material model were, according to ISO527 [38], obtained at small strains (0.05 to 0.25% strain) during the tensile tests. As shown in Figure 11a, however, in the three-point bending tests, strains exceeding 1.5% were apparent. Inspection of the raw stress–strain curves yields that beyond 1% strain, the progression is highly non-linear. Consequently, in future research approaches, considering thickness dependency and orthotropy for elasto-plastic material models should be investigated.

Differences between experiment and simulation could also derive from calculation errors characteristic of the FE method. Errors due to discretization were minimized by performing the mesh convergence study (see Section 2.3.1). The element formulation can also affect the simulation result. However, the utilized Optistruct™ solver is limited to first-order elements for the required geometric non-linear analysis. Furthermore, higher order functions typically reduce element stiffness which, in the present case, would not have resulted in an improvement. Lastly, numerical errors are deemed negligible, since thirteen decimal places were considered in all computations.

Another objective of the present study was to implement a *k*-means clustering algorithm to reduce the number of properties defined by the mapping methodology. Based on an automated approach for the determination of an efficient number of clusters *k*, a model featuring 5 clustered (MAP${}_{k=5}$) was defined, and it had close to identical behavior to that of the fully mapped version (MAP${}_{\mathrm{full}}$) that featured almost 300 material properties. While this only leads to a marginal improvement in the solving time and memory space, the essential benefit of avoiding hundreds of property definitions lies within the usability of the FE model in an industrial context. At this point, it is noteworthy, that the implemented number of five clustered properties is not universally applicable but was computed specifically for the considered structure. Hence, when implementing this methodology for another geometry, a new number of sufficient clusters needs to be determined. Furthermore, the current results suggested that if it is desired to define only one homogeneous material throughout the model, utilization of the *k*-means algorithm to average all apparent thickness and orientation dependent properties (i.e., k=1) yields a superior structural response prediction than employing elastic constants based on experimental data of tensile specimens of the standard 4 mm thickness [38].

Based on the MAP${}_{k=5}$ model, a stochastic analysis was set up. The results showed that the scatter in reaction force inherent in the experiments was accurately reproduced in the numerical simulations for both the horizontal build room aligned sFEA${}_{\mathrm{XZ}}$ and the off-axis sFEA${}_{C}$ parts. Using such an approach in lightweight design diminishes the necessity to define arbitrary safety factors that can lead to over-designed components. Conversely, stochastic analyses allow deduction of probabilistic measures from the structural simulation. For instance, the 95% confidence bounds plotted as shaded areas in Figure 12b indicate that only 5 of the 100 solved models lay outside of it. Consequently, the structural designs can be assessed based on the desired reliability. Thereby, the outcome fundamentally depends on the specified distribution. In the present research, the scatter of elastic parameters, as determined by the tensile tests, was indicated to be normally distributed. However, validity of the chosen function is limited by the relatively small number of samples, namely five specimens per build orientation and thickness. At this point, it cannot be ruled out that higher sample populations would have implied that other parametric functions like the Weibull model could be superior in describing the underlying behavior. In subsequent investigations, larger sample sizes could be considered to verify applicability of the symmetric bell shaped curve utilized in the current study.

A further aspect that needs to be considered is that the probabilistic assessment can only be employed effectively if the simulations capture the fundamental effects occurring in the load case. While the predicted width of the confidence intervals agreed well with the measured one, the simulations did not show the non-linear sigmoid progression apparent during the tests, which led to differences in mean values as exemplarily shown in the detailed view of sFEA${}_{C}$ (see Figure 12b). Due to such deviations, conclusions based on the predicted structural response must be drawn with caution.

Within the scope of the present work, the material modeling and mapping strategy was developed and validated specifically for thin-walled structures represented by 2D shell elements. Nevertheless, it is noteworthy that the procedure can be applied to models consisting of 1D beam elements, since those are typically formulated with thickness information that can also be extracted by the property mapping script. This implies that it can be used to model the thickness-dependent and orthotropic response of lattice structures represented by 1D beam meshes with great detail. However, the computational cost of FE lattice models in which every strut is discretized is very high, which is why the current trend rather goes towards RVE-based homogenization approaches [43,58]. The decision to utilize detailed mapping or homogenization largely depends on the overall size of the FE model and should be justified for every application individually.

Considering thickness dependency and anisotropy in structures discretized by solid elements is a topic for future research. In most commercial FE analysis tools, the directional behavior of solid elements can conveniently be defined by means of a local material coordinate system, which for additively manufactured components, needs to coincide with the build room coordinate system of the machine. While consequently the a priori calculation of element orientation-dependent properties (Equations (6)–(8) is obsolete, the thickness information inherent in 2D shell and 1D beam elements needs to be determined. Thickness analysis in 3D objects can be performed by algorithms that record the diameter of maximum inscribed spheres [59,60]; however, especially in complex structures featuring many junctions, the definition of wall thickness and assignment of corresponding elements remains a challenge for future investigations.

## 5. Conclusions

In this research, a novel material modeling approach for the structural response prediction of components fabricated from orthotropic materials displaying thickness dependency and scatter in mechanical properties was developed based on a shell property mapping strategy proposed by Sindinger et al. [37]. Comparison of numerical simulations obtained from the novel method with standard procedures and experimental tests yielded the following conclusions:

- The thickness dependency of elastic material parameters that was previously reported for various materials fabricated via PBF processes is also inherent in LS PA12 CF polymer matrix composites and needs to be considered for structural simulations of thin-walled components. As previously emphasized by others, considerations of coupon geometries of various thicknesses should be incorporated in ISO and ASTM test standards in order to account for this characteristic inhomogeneous material behavior.
- The developed material modeling approach was deemed suitable to depict the thickness-dependent, orthotropic structural response in deterministic FE analyses by which the simulation accuracy could be improved substantially in comparison to conventional method.
- Using a clustering algorithm, the number of material property definitions necessary to describe the thorough proofreading for typos and grammatical inhomogeneous material behavior could be reduced by two orders of magnitude compared to the initial strategy, in which for each element orientation and thickness, an individual property was generated [37].
- The consideration of scatter in the material modeling regime enabled the prediction of the variability in the reaction force as observed during experiments by means of stochastic FE analysis, which allows estimates regarding the reliability of a design prior to production.

Based on the presented findings, all objectives within the scope of the present work were fulfilled. The disclosed improvement to structural response prediction accuracy allows one to assess thin-walled additively manufactured components at high precision prior to part production, which ultimately paves the way for effective product development processes for functional LS PA12 CF components.

In future research, the inhomogeneous mechanical behavior of PBF materials should not only be considered in assessment of designs but already in structural optimization procedures that command the initial layout of designs [61]. Furthermore, the concept of implementing thickness dependency and anisotropy to structural simulations could be expanded beyond the stiffness question towards failure prediction. Therefore, stochastic FE simulations that additionally account for non-linear elasto-plastic material behavior could also be implemented. This will be investigated in future research. Assuming that the inhomogeneous material behavior is related to the laser scan paths involving variable contour and infill parameter settings (for instance, scan speed and beam offset), it is implied that further research focusing on the optimization of process parameters could improve homogeneity. However, until the inhomogeneous material response is not diminished in commercial PBF systems, advanced modeling and property mapping strategies like that presented in this research are key for valid structural simulations.

## Figures and Tables

**Table 1:** Material parameters determined in the tensile tests given as mean ± standard deviation.

	Young’s Modulus (N/mm^2^)			Poisson’s Ratio		
t **[mm]**	$E_{x}$ **(XY)**	$E_{y}$ **(YZ)**	$E_{z}$ **(ZX)**	$\nu_{\mathrm{xy}}$ **(XY)**	$\nu_{\mathrm{yz}}$ **(YZ)**	$\nu_{\mathrm{zx}}$ **(ZX)**
4	6920 ± 257	3832 ± 147	3114 ± 190	0.392 ± 0.005	0.488 ± 0.004	0.219 ± 0.007
2	5887 ± 66	3387 ± 62	2922 ± 146	0.393 ± 0.005	0.479 ± 0.002	0.212 ± 0.003
1	5549 ± 58	2968 ± 159	2704 ± 148	0.408 ± 0.007	0.445 ± 0.002	0.210 ± 0.004

## Data Availability

The data presented in this study are available on reasonable request from the corresponding author.
